# Supplementary material for: Role and mechanism of NCAPD3 in promoting malignant behaviors in gastric cancer
Source: Front Pharmacol. 2024 Apr 22;15:1341039. doi: 10.3389/fphar.2024.1341039 (PMC11070777; doi:10.3389/fphar.2024.1341039)
Supplement: Supplementary file 11 [file DataSheet2.ZIP › GSEA/immunologic/my_analysis.Gsea.1599462003817/GSE18791_CTRL_VS_NEWCASTLE_VIRUS_DC_4H_UP.html]

Details for gene set GSE18791\_CTRL\_VS\_NEWCASTLE\_VIRUS\_DC\_4H\_UP[GSEA]

|  || Dataset | filtered\_dataset.sample\_info.cls#WT\_versus\_NCAPD3\_MUT |
| Phenotype | sample\_info.cls#WT\_versus\_NCAPD3\_MUT |
| Upregulated in class | WT |
| GeneSet | GSE18791\_CTRL\_VS\_NEWCASTLE\_VIRUS\_DC\_4H\_UP |
| Enrichment Score (ES) | 0.36830783 |
| Normalized Enrichment Score (NES) | 2.0732548 |
| Nominal p-value | 0.0 |
| FDR q-value | 1.0 |
| FWER p-Value | 0.968 |
Table: GSEA Results Summary

  

Fig 1: Enrichment plot: GSE18791\_CTRL\_VS\_NEWCASTLE\_VIRUS\_DC\_4H\_UP      
 Profile of the Running ES Score & Positions of GeneSet Members on the Rank Ordered List

  

| SYMBOL | TITLE | RANK IN GENE LIST | RANK METRIC SCORE | RUNNING ES | CORE ENRICHMENT || 1 | 153364 | MBLAC2 | 79 | 0.838 | -0.0200 | Yes |
| 2 | 55900 | ZNF302 | 145 | 0.740 | -0.0342 | Yes |
| 3 | 11162 | NUDT6 | 162 | 0.716 | -0.0137 | Yes |
| 4 | 57325 | CSRP2BP | 176 | 0.698 | 0.0082 | Yes |
| 5 | 79230 | ZNF557 | 201 | 0.675 | 0.0211 | Yes |
| 6 | 116236 | ABHD15 | 203 | 0.672 | 0.0506 | Yes |
| 7 | 55172 | DNAAF2 | 224 | 0.657 | 0.0655 | Yes |
| 8 | 146050 | ZSCAN29 | 226 | 0.654 | 0.0942 | Yes |
| 9 | 51250 | C6orf203 | 230 | 0.650 | 0.1213 | Yes |
| 10 | 84816 | RTN4IP1 | 232 | 0.649 | 0.1498 | Yes |
| 11 | 84078 | KBTBD7 | 295 | 0.594 | 0.1313 | Yes |
| 12 | 81889 | FAHD1 | 299 | 0.592 | 0.1557 | Yes |
| 13 | 27079 | RPUSD2 | 320 | 0.582 | 0.1673 | Yes |
| 14 | 8125 | ANP32A | 326 | 0.578 | 0.1897 | Yes |
| 15 | 9786 | KIAA0586 | 379 | 0.547 | 0.1763 | Yes |
| 16 | 9815 | GIT2 | 394 | 0.540 | 0.1904 | Yes |
| 17 | 79896 | THNSL1 | 407 | 0.534 | 0.2056 | Yes |
| 18 | 10885 | WDR3 | 430 | 0.515 | 0.2127 | Yes |
| 19 | 22796 | COG2 | 445 | 0.507 | 0.2253 | Yes |
| 20 | 54482 | TRMT13 | 449 | 0.506 | 0.2459 | Yes |
| 21 | 10940 | POP1 | 497 | 0.474 | 0.2329 | Yes |
| 22 | 54920 | DUS2 | 509 | 0.470 | 0.2460 | Yes |
| 23 | 29090 | TIMM21 | 537 | 0.453 | 0.2467 | Yes |
| 24 | 2188 | FANCF | 545 | 0.449 | 0.2618 | Yes |
| 25 | 9416 | DDX23 | 553 | 0.445 | 0.2767 | Yes |
| 26 | 9816 | URB2 | 581 | 0.434 | 0.2765 | Yes |
| 27 | 93134 | ZNF561 | 590 | 0.431 | 0.2900 | Yes |
| 28 | 55039 | TRMT12 | 599 | 0.424 | 0.3033 | Yes |
| 29 | 56339 | METTL3 | 613 | 0.417 | 0.3125 | Yes |
| 30 | 5147 | PDE6D | 624 | 0.410 | 0.3237 | Yes |
| 31 | 26953 | RANBP6 | 630 | 0.406 | 0.3383 | Yes |
| 32 | 51287 | COA4 | 660 | 0.396 | 0.3349 | Yes |
| 33 | 78996 | C7orf49 | 661 | 0.396 | 0.3527 | Yes |
| 34 | 56252 | YLPM1 | 665 | 0.394 | 0.3683 | Yes |
| 35 | 5987 | TRIM27 | 695 | 0.380 | 0.3642 | No |
| 36 | 6182 | MRPL12 | 729 | 0.359 | 0.3563 | No |
| 37 | 27300 | ZNF544 | 759 | 0.343 | 0.3505 | No |
| 38 | 26608 | TBL2 | 821 | 0.304 | 0.3196 | No |
| 39 | 6732 | SRPK1 | 835 | 0.291 | 0.3232 | No |
| 40 | 55148 | UBR7 | 987 | -0.396 | 0.2307 | No |
| 41 | 5889 | RAD51C | 1059 | -0.451 | 0.1991 | No |
| 42 | 27250 | PDCD4 | 1170 | -0.537 | 0.1429 | No |
| 43 | 57533 | TBC1D14 | 1304 | -0.708 | 0.0775 | No |
Table: GSEA details [plain text format]

  

Fig 2: GSE18791\_CTRL\_VS\_NEWCASTLE\_VIRUS\_DC\_4H\_UP      
 Blue-Pink O' Gram in the Space of the Analyzed GeneSet

  

Fig 3: GSE18791\_CTRL\_VS\_NEWCASTLE\_VIRUS\_DC\_4H\_UP: Random ES distribution      
 Gene set null distribution of ES for **GSE18791\_CTRL\_VS\_NEWCASTLE\_VIRUS\_DC\_4H\_UP**
